# Influence of the 5-HT3A Receptor Gene Polymorphism and Childhood Sexual Trauma on Central Serotonin Activity

**DOI:** 10.1371/journal.pone.0145269

**Published:** 2015-12-23

**Authors:** Kuk-In Jang, Seung-Hwan Lee, Hyu Jung Huh, Jeong-Ho Chae

**Affiliations:** 1 Department of Biomedicine & Health Sciences, The Catholic University of Korea, College of Medicine, Seoul, Korea; 2 Department of Psychiatry, Inje University College of Medicine, Ilsan Paik Hospital, Goyang, Korea; 3 Clinical Emotion and Cognition Research Laboratory, Inje University, Goyang, Korea; 4 Department of Psychiatry, Seoul St. Mary's Hospital, The Catholic University of Korea, College of Medicine, Seoul, Korea; Radboud University Medical Centre, NETHERLANDS

## Abstract

**Background:**

Gene-environment interactions are important for understanding alterations in human brain function. The loudness dependence of auditory evoked potential (LDAEP) is known to reflect central serotonergic activity. Single nucleotide polymorphisms (SNPs) in the 5-HT3A serotonin receptor gene are associated with psychiatric disorders. This study aimed to investigate the effect between 5-HT3A receptor gene polymorphisms and childhood sexual trauma on the LDAEP as an electrophysiological marker in healthy subjects.

**Methods:**

A total of 206 healthy subjects were recruited and evaluated using the childhood trauma questionnaire (CTQ) and hospital anxiety and depression scale (HADS). Peak-to-peak N1/P2 was measured at five stimulus intensities, and the LDAEP was calculated as the linear-regression slope. In addition, the rs1062613 SNPs of 5-HT3A (CC, CT, and TT) were analyzed in healthy subjects.

**Results:**

There was a significant interaction between scores on the CTQ-sexual abuse subscale and 5-HT3A genotype on the LDAEP. Subjects with the CC polymorphism had a significantly higher LDEAP than T carriers in the sexually abused group. In addition, CC genotype subjects in the sexually abused group showed a significantly higher LDAEP compared with CC genotype subjects in the non-sexually abused group.

**Conclusions:**

Our findings suggest that people with the CC polymorphism of the 5-HT3A gene have a greater risk of developing mental health problems if they have experienced childhood sexual abuse, possibly due to low central serotonin activity. Conversely, the T polymorphism may be protective against any central serotonergic changes following childhood sexual trauma.

## Introduction

Childhood trauma appears to be a crucial etiological factor in the development of many serious mental and behavioral disorders across the lifespan [1]. Epidemiological studies indicate that children exposed to early adverse experiences have an increased risk of developing depression and/or anxiety disorders. Early life stress can persistently sensitize central nervous system (CNS) circuits that are integrally involved in regulating stress and emotion, and this mechanism may be an underlying biological substrate of increased vulnerability to subsequent stress and the development of depression and anxiety [2].

Childhood sexual abuse (CSA) has been extensively studied and has been suggested its linkage with health problems and mental illnesses [3–6]. Individuals who have experienced CSA show an increased prevalence of post-traumatic stress disorder (PTSD), which has been reported in 37%–53% of cases [7]. CSA is also associated with chronic fatigue, psychosis, anxious attachment, and depression [8–10]. Girls are more exposed to sexual abuse than boys and have an increased suicide attempt rate [11]. In addition, CSA can cause sexual dysfunction and adult sexual trauma by re-victimization [12–14]. Individuals with CSA also exhibit psychosocial problems such as a lack of self-esteem and lower social support and positive self-worth [15]. Additionally, women with a history of CSA are more likely to present with hypothalamic-pituitary-adrenal axis dysfunction [16].

Gene-environment interactions have been extensively studied to understand the underlying pathophysiology of psychiatric disorders. Bellani et al. [17] reported a significant interaction among childhood physical and sexual abuse and several genetic polymorphisms associated with the development of psychiatric disorders in adulthood. The rs1062613 single-nucleotide polymorphism (SNP) C178T in the upstream regulatory region of the 5-HT3A gene was identified to be functionally important in psychiatric disorders [18]. Iidaka et al. [19] found greater activity in the amygdala and dorsal and medial prefrontal cortices during the face recognition test in healthy subjects with the CC allele compared to those with the CT allele. CC carriers also had faster reaction times. When CC and T carriers are exposed to stress, the former exhibit a loss of gray matter in the hippocampus [20], elevated emotion-elicited heart rate, and high alpha band activity in the right frontal area on electroencephalography (EEG) recordings, all of which are correlates of major depression [21]. In addition, the CC genotype is associated with increased anxiety and amygdala hyper-responsiveness in patients with irritable bowel syndrome [22]. This polymorphism may affect the personality trait of harm avoidance in women; TT carriers have lower ratings on harm avoidance and nonconformity [23]. Nonetheless, there are null associations between major depression in women and haplotype blocks in the 5-HT3A gene in 180 unrelated patients with mood disorders [24].

The loudness dependence of auditory evoked potential (LDAEP) is considered a electrophysiologic biomarker of central serotonergic neurotransmission [25] and a possible marker of serotonin transmission; a high LDAEP reflects low central serotonergic neurotransmission and *vice versa* [25, 26]. Previous studies have shown that the LDAEP is closely related to altered serotonin levels in patients with mood and anxiety disorders [27–35]. It has also been suggested as a marker of serotoninergic function in patients with schizophrenia [34, 36]. In addition, The LDAEP is associated with brain-derived neurotrophic factor (BDNF) gene polymorphisms in healthy subjects [37].

In this study, we aimed to investigate the interaction between rs1062613 SNPs of the 5-HT3A receptor gene polymorphism and CSA on the LDAEP, an possible electrophysiological marker for serotonin activities. We hypothesized that there would be a significant interaction between CSA and 5-HT3A genotype on the LDAEP. Second, among subjects who experienced CSA, those with the CC genotype would produce a higher LDAEP compared with T carriers. Third, T carriers would exhibit preserved serotonin activity as indicated by a low LDAEP.

## Methods

### Subjects

The subjects were 206 healthy adults (110 men and 96 women). The mean age was 24.05 ± 3.25 years (range: 19–32 years). All subjects were recruited through advertisements in local newspapers. They were native Koreans with Korean parents. Subjects were invited for a comprehensive interview that included the Structured Clinical Interview for the Diagnostic and Statistical Manual of Mental Disorders, Fourth Edition (SCID I and SCID II) to exclude current and/or lifetime Axis I and II psychiatric disorders [38, 39]. Subjects with hearing problems, organic brain disease, left-handedness, any history of cigarette smoking within a year of the study time-point, or family history of mental disorders were excluded. The study was approved by the Institutional Review Board of Seoul Saint Mary’s Hospital, College of Medicine, The Catholic University of Korea. All subjects provided signed informed consent.

### Electrophysiological assessment

To avoid any possible hormonal effects on the LDAEP, measurements in female subjects were taken during the 2^nd^–5^th^ day of menstruation [40]. Participants were seated in a comfortable chair in a sound-attenuated room. The auditory stimulation comprised 1000 stimuli with an interstimulus interval randomized to 500–900 ms. Tones of 1000 Hz and 80-ms duration (with a 10-ms rise and fall) were presented at five intensities (55, 65, 75, 85, and 95 dB SPL) via headphones (MDR-D777, Sony, Tokyo, Japan). These stimuli were generated by the E-Prime software (Psychology Software Tools, Pittsburgh, PA, USA). EEG data were recorded from 32 scalp sites using silver/silver-chloride electrodes according to the International 10–20 system (impedance, 5 kΩ) using an Auditory Neuroscan NuAmp amplifier (Compumedics USA, El Paso, TX, USA). Data were collected at a sampling rate of 1000 Hz using a bandpass filter of 0.5–100 Hz. In addition, four electrodes were used to measure both horizontal and vertical electrooculograms. The ground and reference electrodes were placed on the forebrain and bilateral mastoids, respectively. Data were reanalyzed using Scan 4.5 software with a bandpass filter of 1–30 Hz, and ocular contamination was removed using standard blink correction algorithms [41]. Event-related potential sweeps with artifacts exceeding 70 μV were rejected at all electrode sites. For each intensity and participant, the N1 (most negative amplitude 80–130 ms after the stimulus) and P2 peak (most positive amplitude 130–230 ms after the stimulus) were determined at the Fz, Cz, Pz, C3, and C4 electrodes. The peak-to-peak N1/P2 amplitudes were calculated for each stimulus intensity, and the LDAEP was calculated by the slope of the linear regression [26]. The mean LDAEP value (averaged from the Fz, Cz, C3, C4, and Pz) was used in further analysis.

### Rating scale

#### Hospital anxiety and depression scale

Anxiety and depression symptoms were assessed by the Hospital anxiety and depression scale (HADS) [42], a self-report, 14-item questionnaire that is composed of two 7-item subscales of anxiety and depression. Each item is answered on a 4-point (0–3) response category and the possible scores of the two subscales range from 0–21.

#### Childhood trauma questionnaire

We assessed traumatic childhood experiences using the short form of the childhood trauma questionnaire (CTQ). The CTQ-short form is a self-report questionnaire consisting of 28 items (25 clinical and 3 validity items) [43]. It measures five categories of childhood maltreatment including emotional, physical neglect, emotional, physical, and sexual abuse. Each subscale has 5 items with a 5-point frequency of occurrence, and subscale scores range from 5–25. For clinical samples, researchers have usually used the moderate to severe cut-off scores for each subscale to classify subjects as positive for a history of childhood trauma in that category. The cut-off scores are ≥10, ≥13, ≥8, ≥10, and ≥15 for physical abuse, emotional abuse, sexual abuse, physical neglect, and emotional neglect, respectively [44, 45]. However because the cut-offs are too high for the normative sample of the present study, we used lower cut-off scores: ≥8 for physical abuse, ≥11 for emotional abuse, ≥6 for sexual abuse, ≥8 for physical neglect, and ≥13 for emotional neglect. Although the distributions of CTQ scores differ according to age and sex, these cut-off scores were higher than the 90^th^ percentiles of data from the previous community sample study [46].

### Genotyping

We collected blood samples (5–10 ml) in EDTA tubes, and genomic DNA was isolated using a NucleoSpin Blood DNA Extraction Kit (Macherey-Nagel, Düren, Germany) according to the manufacturer’s instructions. Genotyping was performed by high-resolution melting (HRM) curve analysis. Polymerase chain reaction (PCR) was performed in a 20-μl reaction volume using the 96-well Bio-Rad CFX96 Real time PCR system (Bio-Rad, Hercules, CA, USA). Reaction mixtures included 1.5 μl of genomic DNA as a template, 200 mM of each primer for the rs1062613 SNP of HTR3A (forward 5′-CAT GAG GTT GGC AGA GGG-3′; reverse 5′-TCC CGA AGT CTG CTT ACC-3′; BMS, Daejeon, South Korea), 1× Sso Fast EvaGreen SuperMix (Bio-Rad), and sterile H_2_O. The amplification protocol started with a 98°C step for 3 min followed by 39 cycles of 98°C for 10 s and 58°C for 20 s. After an initial step of 95°C for 10 s and 65°C for 10 s, melting curves were generated from 65–95°C in increments of 0.3°C/cycle. Melting profiles were analyzed with Bio-Rad Precision Melt software.

The genotype frequencies of CC, CT, and TT polymorphisms in the 5-HT3A SNPs were 55.8% (n = 115), 39.3% (n = 81), and 4.9% (n = 10), respectively. The group size of the TT genotype (n = 10) was too small to examine between-subject effects [21]. The allele frequencies are in line with previous studies that concluded that the T allele is less frequent than the C allele in European and Asian populations [47, 48]. Hammer et al. reported that genotype frequencies were CC (n = 2,555), CT (n = 1,369), and TT (n = 202) in European subjects [47]. Kang et al. described the following 5HTR3A C178T (rs1062613) genotype groups in Korean subjects with schizophrenia: CC (n = 146), CT (n = 110), and TT (n = 24) [48]. Therefore, T (CT and TT, n = 91) and CC (n = 115) carrier groups were categorized for further statistical analysis.

### Statistical analyses

We compared demographic variables using χ^2^ and independent *t*-tests. A repeated measures analysis of variance (ANOVA) was used independently with subscales of childhood trauma (yes *vs*. no) and genotype (CC *vs*. T carriers) as between-subject factors; the outcome variables were LDAEP’s five electrodes (Fz, Cz, C3, C4, and Pz) as within-subject factors. Gender and age were considered as covariates. The mean LDAEP value was the outcome variable in the absence or presence of childhood trauma and in CC and T carriers. We examined the between-subject effect in post hoc analyses by univariate ANOVA with age and gender as covariates. Statistical significance was set at *p* < 0.05 (two-tailed). Multiple tests were corrected by the Bonferroni method, and *p* < 0.01 was considered significant. The power of genetic association was calculated by using G*Power 3.1.7 software [49].

## Results

There were no significant differences in age, education, sex, or scores on two subscales of the HADS or five CTQ categories between CC and T carriers (Table 1).

**Table 1:** Characteristics of the study population according to 5-HT3A genetic polymorphism.

*Variables*		CC(n = 115)	Tcarrier(n = 91)	Genotype comparison	
				t or *χ*2	P-value
Age (years)		23.90 ± 3.53	24.24 ± 3.13	0.74	0.461
Sex (male/female)		60/55	50/41	0.16	0.692
Education (years)		14.70 ± 2.02	14.74 ± 1.74	0.15	0.879
HADS					
	Anxiety	5.32 ± 3.08	5.32 ± 3.45	0.01	0.995
	Depression	4.97 ± 1.76	5.42 ± 2.25	1.54	0.124
CTQ					
	Physical abuse	8.16 ± 3.32	8.13 ± 3.40	0.52	0.958
	Emotional abuse	6.47 ± 2,71	6.89 ± 3.02	1.05	0.294
	Sexual abuse	5.97 ± 1.90	5.70 ± 1.69	1.03	0.304
	Physical neglect	7.23 ± 2.36	6.88 ± 2.20	1.11	0.270
	Emotional neglect	9.81 ± 4.15	9.62 ± 4.20	0.32	0.751

HADS, hospital anxiety and depression scale; CTQ, childhood trauma questionnaire

**Fig 1:**
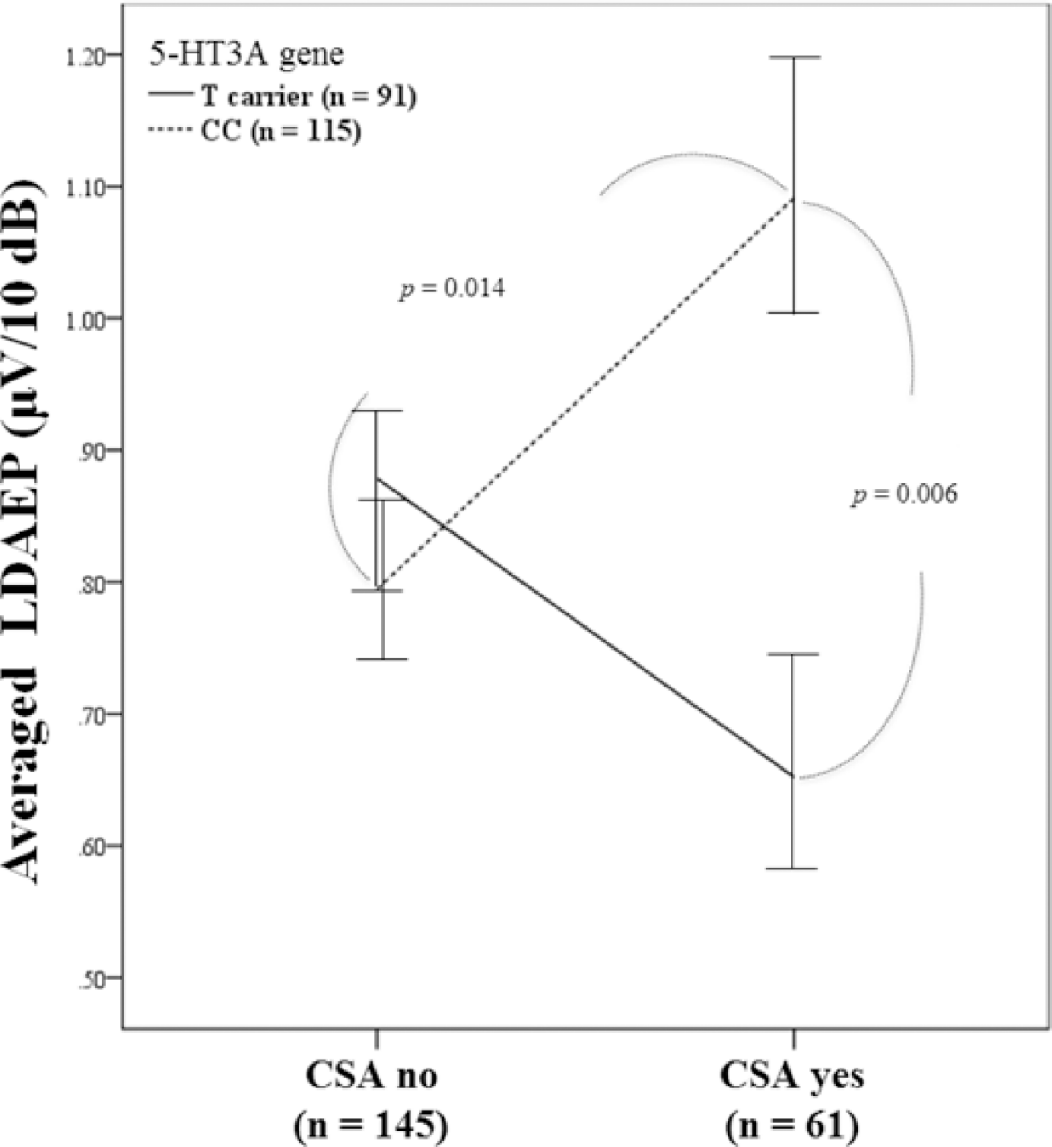
The effect of the interaction between CSA and 5-HTR3A gene polymorphism on averaged LDAEP.

Sexual abuse showed a significant gene-environmental interaction as measured by repeated measures ANOVA (Table 2). There was a significant interaction between CSA and 5-HT3A polymorphism on LDAEP (*F* = 7.101, df = 1, *p* = 0.008; Fig 1) (Bonferroni-corrected *p* < 0.01).

**Table 2:** Between-subject effect of repeated measures ANOVA for each category of the childhood trauma questionnaire (CTQ) on LDAEP (Fz, Cz, C3, C4, and Pz) as outcome variables.

CTQ Category(Yes, %)	CC(n = 115)	Tcarrier(n = 91)	F	df	P-value
Physical abuse	50.4%	42.9%	2.738	1	0.100
Emotional abuse	9.6%	11.0%	3.010	1	0.084
Sexual abuse	31.3%	27.5%	7.101	1	**0.008**
Physical neglect	38.3%	30.8%	0.009	1	0.924
Emotional neglect	25.2%	24.1%	0.075	1	0.785

CTQ, childhood trauma questionnaire; LDAEP, loudness dependence of auditory evoked potential

**Fig 2:**
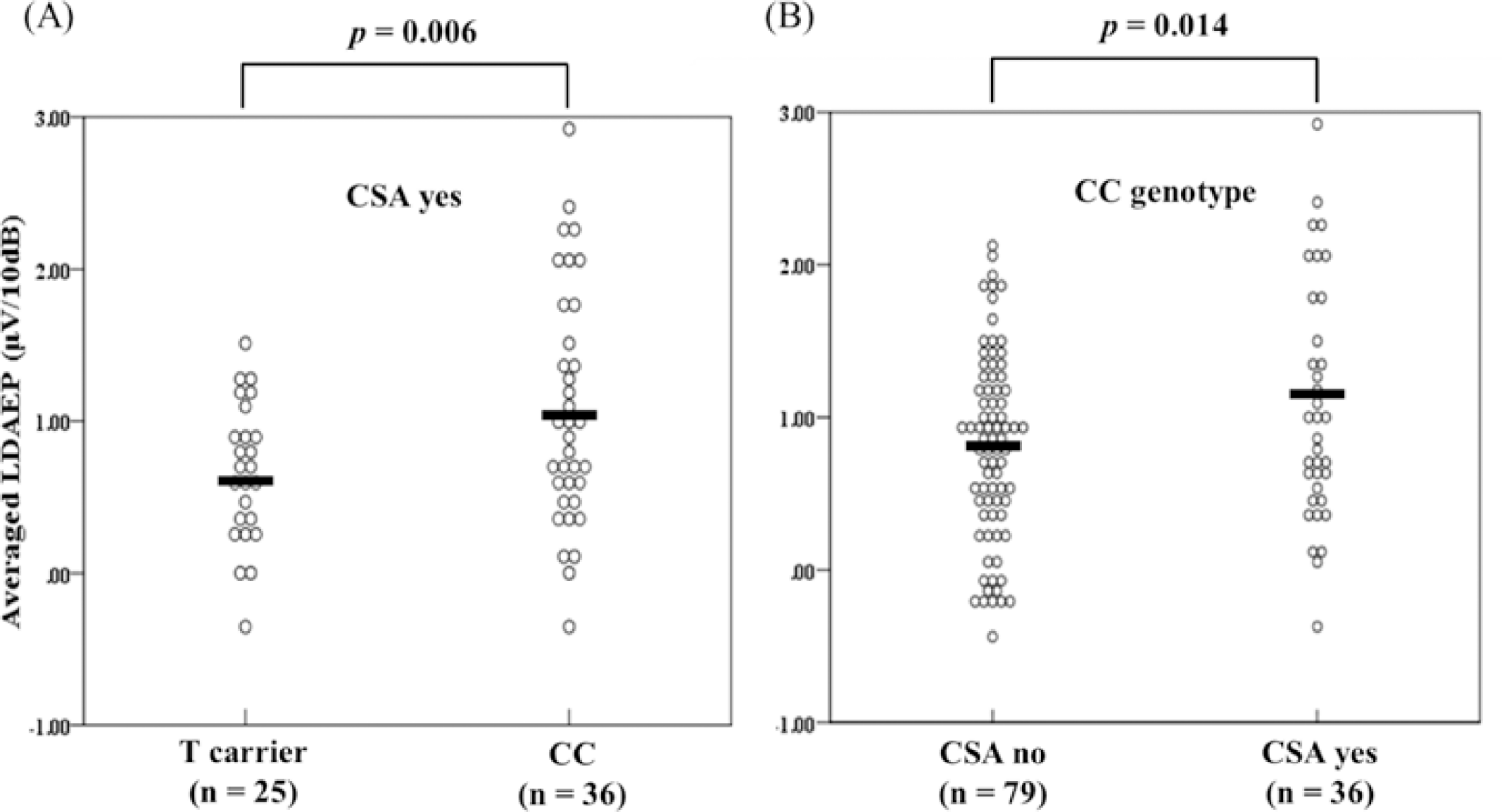
Results of univariate comparison of averaged LDAEP values between T carriers and CC genotype of the 5-HTR3A gene in people with CSA (A). With and without CSA among CC genotype subjects (B). Covariates were gender and age.

The post hoc analysis revealed a significantly higher mean LDAEP in the CC carriers compared with the T carriers in the CSA group (*F* = 8.170, *d*.*f*. = 1, *p* = 0.006; Fig 2A). Within the CC genotype group, the LDAEP was significantly higher in subjects who experienced CSA compared with those who did not (*F* = 6.278, *d*.*f*. = 1, *p* = 0.014; Fig 2B).

There were no significant gene-environmental interactions associated with physical/emotional abuse or physical/emotional neglect.

The power of genetic association was calculated for sexual abuse category because it had a significant interaction. The power was 0.77 to detect an effect size of 0.19 in the genotype and allele frequency analyses.

## Discussion

In this study, we examined the gene-environment interaction between childhood trauma and 5-HTR3A gene polymorphisms on the LDAEP, a biological correlate of central serotonin activity. We observed a significant gene-environment interaction; a history of CSA significantly interacted with 5-HT3A polymorphism to affect the LDAEP value. Among those who had experienced CSA, CC carriers had an increased LDAEP compared with T carriers. Furthermore, the LDAEP was higher in subjects who had experienced CSA versus those who had not in the CC genotype group.

The major finding of the present study was that a history of CSA was significantly involved in the interaction between 5-HT3A polymorphism status and LDAEP value. This significant interaction was only present in those with a history of childhood sexual abuse, not other types of childhood trauma. Previous studies have revealed that sexual trauma has a greater biopsychosocial impact than other types of child abuse and neglect. Sheffield et al. [50] investigated the association between childhood abuse types and auditory hallucinations in patients with psychotic disorders and control subjects. They found that subjects with auditory hallucinations were significantly associated with CSA. In the absence of sexual abuse, emotional and physical abuse was not related to a higher rate of auditory hallucinations. Subica [51] found that CSA exposure had a significant impact on PTSD and depression compared with exposure to physical abuse. They showed that CSA uniquely predicts PTSD, depression, and physical health problems; therefore, the presence of CSA adversely affects mental illnesses compared with other types of child abuse and neglect. These previous findings are in line with our results.

Among subjects who had experienced childhood sexual trauma, we found that LDAEP was higher in subjects with a CC genotype than in T carriers. These results suggested that serotonin function is significantly influenced by gene-environmental interactions. Several studies have provided evidence that an interaction between CSA and serotonin gene polymorphisms is related to mental illness. Cicchetti et al. [52] found that sexual abuse and the 5-HTT short/short allele genotype predicts higher rates of depression, anxiety, and somatic symptoms. Aguilera et al. [53] reported that CSA has a greater impact on depressive symptoms in Met allele carriers of the BDNF gene and S carriers of the 5-HTTLPR polymorphism. Furthermore, Fisher et al. [54] found an interaction between the 5-HTTLPR polymorphism and childhood maltreatment on recurrent depressive disorder.

In addition, 5-HT3A is known to interact with childhood trauma in the development of various psychopathologies. The 5-HT3A receptor is a cation-selective ion channel expressed in the amygdala, hippocampus, and caudate [18]. Gatt et al. [20] investigated the effect of 5-HT3A gene polymorphisms and early trauma on brain networks and depressed mood in 397 healthy subjects and observed a significant reduction in hippocampal gray matter in CC genotype subjects compared with T carriers. Additionally, the interaction between 5-HT3A SNP status and childhood maltreatment predicts a depressed mood. Another study reported that individuals with both BDNF methionine and HTR3A CC genotypes and early life stress exposure demonstrated elevated emotion-elicited heart rate and right frontal hyper-activation with right parietotemporal hypoactivation on EEG [21]. They interpreted this as a brain-arousal profile indicative of a higher risk of developing depression. Iidaka et al. [19] showed greater activity in the amygdala and dorsal and medial prefrontal cortices during a face-recognition test in healthy subjects with the CC genotype compared to those with a CT genotype; subjects with a CC genotype also had faster reaction times. However, studies in Asian population have yielded inconsistent results; in Japanese subjects, major depression showed an association with 5-HT3B but not 5-HT3A gene polymorphisms [24].

Compare with those who had no experienced CSA, subjects with the CC genotype had a higher LDAEP if they had experienced CSA. A high LDAEP can be interpreted as low central serotonergic activity in the brain [26]. In their single-photon emission computed tomography study, Lee et al. [55] found that healthy controls with a higher LDAEP showed low serotonin transporter availability that is (indicative of low serotonin tone). Low serotonergic activity in patients with major depressive disorder has been associated with suicide attempts [56–58] and more severe somatic symptoms of depression, such as a loss of appetite, insomnia, and sexual dysfunction [59]. It was recently hypothesized that serotonin plays a role in behavioral inhibition [60]. Evidence from an animal study suggests the involvement of serotonin depletion in a failure of response inhibition [61]. Collectively, these findings indicate that people with the 5HT3A CC genotype may show increased impulsivity and have a higher suicide risk when they have history of childhood sexual trauma.

### Limitation

It should be considered that the samples in the present study were healthy subjects without any mental disorders, so there were no symptomatic differences among the subgroups. Moreover, we could not confirm any pathological characteristics between subjects with low and high LDAEPs when CSA was excluded as a variable. The results of this study could explain the interaction between CSA and 5-HT3A on serotonin function in the general population. However, the CTQ may not precisely reflect the participants’ traumatic childhood experiences because it is subjective and a retrospective self-report. Future clinical studies should be conducted with a large sample size and consider genetic characteristics corresponding to mental illness prevalence in the gene-environmental model. Furthermore, investigations should compare the magnitudes of the averaged LDAEPs in individuals who experienced CSA and subjects with the CC genotype to patient populations.

### Conclusions

The results of this study confirmed that LDAEP magnitude is clinically useful to indicate the Gene × Environment effect. The mean LDAEP value of the CC + CSA group in the present study was similar to depressed patients who had made suicide attempts in an earlier investigation [29]. The results were similar in healthy *vs*. healthy and healthy *vs*. schizophrenia in a case-control study [36] (S2 Table). The present study suggests that the CC genotype could be a genetic risk factor for a higher LDAEP, reflecting low central serotonergic function. Conversely, the T allele may play a role in preventing psychophysiological conditions in subjects who have experienced CSA.

## Supporting Information

S1 Table Raw data. (PDF)

S2 Table The mean LDAEP magnitude in comparison with previous studies. (DOCX)
