# Supplementary material for: Influence of the 5-HT3A Receptor Gene Polymorphism and Childhood Sexual Trauma on Central Serotonin Activity
Source: PLoS One. 2015 Dec 23;10(12):e0145269. doi: 10.1371/journal.pone.0145269 (PMC4689356; doi:10.1371/journal.pone.0145269)
Supplement: S1 Table — (PDF) [file pone.0145269.s001.pdf]

S1 Table. Raw data

Age	Educations	Gender	LDAEP					HT3A	Anxiety	Depression	Physical Abuse	Emotional Abuse	Sexual Abuse	Emotional Neglect	Physical Neglect
			Fz	Cz	C3	C4	Pz								
30	18	m	0.3068	0.71043	0.1024	0.71009	0.57506	C/C	1	3	9	6	5	6	5
29	18	m	1.00266	1.43576	1.06875	0.82272	0.48363	C/C	3	3	9	7	5	11	6
31	18	m	0.87161	0.89929	0.76906	1.74298	0.64694	C/C	1	2	10	7	9	11	5
22	16	m	-0.2777	0.79405	1.54847	2.95761	-0.24908	C/C	3	7	5	5	5	8	5
22	16	m	2.42618	2.21187	1.94688	1.48305	1.37145	C/C	7	6	8	7	5	5	5
23	15	m	1.65264	1.04254	1.64339	0.90988	0.48641	C/C	6	5	5	5	5	5	5
22	15	m	0.49608	1.21751	1.13443	0.542	1.07728	C/C	4	3	10	5	6	5	9
29	18	m	1.37114	-0.1938	-0.24158	-0.16945	0.00953	C/T	4	8	5	5	5	7	6
23	12	m	-1.27413	1.66974	0.79317	1.430697	-0.00319	C/C	9	5	15	5	5	11	5
28	16	m	0.88017	0.23282	-0.2504	0.42794	-0.24411	C/T	1	4	7	5	5	8	9
23	16	m	1.20043	1.06655	-0.11371	1.01807	0.17388	T/T	7	5	9	8	5	5	7
23	15	m	-0.27201	0.35216	0.02784	0.20929	0.16485	C/C	2	3	5	5	6	5	7
24	12	m	0.48619	1.57337	1.11009	0.46874	0.15772	C/C	7	4	10	5	5	5	6
25	17	m	-0.00284	0.31412	0.16487	0.15356	-0.00749	C/C	5	7	6	6	8	8	5
20	12	m	1.0204	-0.20654	-0.35029	0.59144	0.51807	C/T	4	4	6	9	5	5	8
25	16	m	0.52792	0.46347	0.5483	0.51785	0.16913	T/T	6	5	9	5	5	5	5
21	13	m	0.64824	1.32804	0.93464	0.65515	1.29068	C/C	5	4	8	6	5	13	7
21	13	m	1.84879	1.49573	1.60832	1.75217	-0.10744	C/C	7	4	9	6	5	9	7
20	13	m	1.22702	1.32028	1.38191	0.99668	0.44147	C/T	1	3	6	5	5	5	5
27	16	m	1.03416	0.96877	-0.1408	0.67522	0.17413	C/T	4	4	7	5	5	11	7
20	12	m	0.87939	1.33484	1.10833	1.14998	0.93026	C/T	8	7	9	5	5	15	5
27	18	m	-0.56903	-0.31788	0.01277	0.03689	0.24305	C/C	3	4	11	5	5	11	9
21	14	m	1.27181	1.71664	1.60285	1.75897	1.44309	C/T	6	6	5	5	5	5	13
23	13	m	0.39367	0.1478	0.14414	-0.21001	-0.06794	C/C	3	6	5	5	5	5	5
31	12	m	0.62091	1.09828	-0.04194	1.08358	0.7143	C/T	6	8	7	7	5	17	9
20	14	m	-0.16548	0.42472	-0.47164	-0.28406	-0.34577	C/T	10	7	8	5	5	7	7
20	13	m	0.02844	1.14817	0.30288	1.07674	0.95053	C/T	0	4	5	5	5	10	7
23	13	m	-0.27236	0.47027	-0.36274	-0.11769	-0.14236	T/T	7	6	7	13	5	8	9
25	15	m	0.02248	0.24325	0.4542	0.10292	0.30669	C/C	2	3	5	5	5	7	6
28	16	m	-0.09762	1.70934	0.31108	1.33514	0.51447	C/T	3	3	5	5	5	6	5
19	12	m	0.42566	0.65056	0.92587	0.77112	0.40637	C/C	7	6	9	7	5	13	6
21	12	m	-0.12505	0.8829	0.86549	1.05393	0.90817	C/C	5	4	16	11	11	17	12
21	13	m	1.67555	1.12333	1.33475	1.38383	0.96207	C/T	7	4	10	5	9	14	7
20	12	m	1.41392	1.82392	1.71219	1.73458	1.05723	T/T	3	4	7	5	5	5	9
20	13	m	1.17039	0.43722	0.33722	0.51785	0.58863	C/T	4	2	8	5	5	7	5
22	13	m	1.73475	2.80871	1.78967	1.61924	0.08472	C/T	2	2	5	5	5	5	5
19	12	m	-0.28588	-0.52496	1.012	-0.33314	0.24379	C/C	5	4	14	8	10	16	9
20	12	m	1.04825	0.7828	-0.07334	-0.07952	0.16692	C/C	4	2	7	5	8	10	13
20	12	m	0.60855	1.25573	0.91009	1.071462	0.76809	C/C	3	4	5	5	5	7	9
26	14	m	0.34698	1.11952	0.96795	0.37301	0.77751	C/T	1	5	7	10	11	10	7
28	15	m	0.46742	0.62882	0.06429	0.62852	0.52705	C/C	6	6	11	6	9	13	11
28	12	m	0.14094	0.62921	0.73153	1.11705	1.48832	C/T	3	6	5	5	5	8	5
20	12	m	1.50012	1.28523	1.07786	1.23923	0.51476	C/C	9	7	7	5	5	8	9
20	13	m	0.67369	0.26643	0.38803	0.53649	0.6467	C/C	2	3	8	10	5	18	7
31	16	m	0.5936	1.91795	1.44931	2.44236	1.04777	T/T	7	9	6	5	5	7	8
29	16	m	0.64572	0.79894	0.11611	1.1333	0.9353	C/C	1	5	9	5	7	9	11
29	16	m	1.74247	2.19184	1.87745	1.8815	1.09303	C/C	6	5	6	5	6	5	6
32	16	m	2.63244	1.73435	1.83437	0.74955	1.13647	C/T	4	3	5	5	5	6	5
30	16	m	0.14038	1.07158	0.38482	0.78488	0.45997	C/C	5	6	9	5	8	8	6
23	14	m	0.32422	0.42854	0.49672	0.29451	0.31928	C/T	6	5	8	5	5	6	5
23	14	m	1.47339	1.79857	2.23839	2.47457	0.55878	C/T	3	5	7	5	5	9	6
19	13	m	1.46002	0.86771	0.74818	2.14415	1.09751	C/C	7	9	6	6	5	17	8
21	13	m	0.58177	1.0892	0.78001	1.04156	0.80709	C/T	2	4	5	5	5	12	8
25	16	m	1.81184	1.7864	1.16467	2.0279	1.05979	C/T	3	3	5	5	5	16	5
28	16	m	0.7585	0.79284	0.62639	1.06134	0.54702	C/T	8	4	6	9	6	10	5
31	16	m	1.10021	1.68675	1.23665	1.32971	0.95705	C/C	3	6	13	6	7	9	10
31	17	m	0.44959	0.76712	0.51934	0.70939	0.20426	C/C	0	4	8	6	5	11	6
30	16	m	0.87328	0.97485	1.08227	0.78305	0.47337	C/T	8	6	15	7	6	10	7
25	15	m	0.64117	1.69019	0.75766	0.91777	1.01782	C/C	5	3	5	5	5	5	5
25	14	m	0.61286	0.61224	0.53964	0.38862	0.54961	C/T	3	6	12	10	5	14	6
23	15	m	0.04257	0.81394	0.23914	0.59831	0.44136	C/C	3	5	23	11	6	15	13
25	15	m	1.42872	1.73016	1.45226	1.43446	0.68426	C/C	6	2	8	5	12	5	5
23	16	m	0.64534	1.00543	0.11153	0.62005	0.18443	C/T	7	6	7	6	5	12	7
26	15	m	0.964	0.56926	0.58376	0.50808	0.7876	C/T	6	7	6	5	10	8	6
21	14	m	0.10755	2.30699	1.04535	1.35589	1.22177	C/C	7	6	12	7	6	15	8
23	16	m	0.21589	-0.13025	-0.18811	-0.11883	0.1842	C/T	8	4	12	5	5	6	8
26	15	m	0.55115	0.39858	0.19545	0.27847	0.42853	C/T	7	7	13	11	6	12	9
26	12	m	-0.367542	0.700006	2.24513	0.06192	1.46347	C/C	1	6	9	5	5	10	7
28	16	m	0.42875	0.26476	0.32052	0.15028	-0.1741	C/C	3	8	5	5	5	10	5
23	12	m	0.90214	1.04803	0.41469	1.1329	0.72512	C/T	5	6	13	21	5	18	11
24	14	m	-0.07113	3.74329	-0.02375	0.83528	2.42054	C/C	3	5	14	13	15	13	15
28	13	m	0.22747	0.59085	2.01096	1.2808	0.51115	C/C	6	4	11	11	5	15	9
22	14	m	1.46501	1.53325	1.11304	0.58924	0.23164	C/C	6	6	6	5	5	9	5
22	12	m	1.68106	1.01374	0.66101	1.20418	-0.00615	C/C	2	4	5	5	5	6	6
26	14	m	-0.58267	-0.51938	-0.28247	-0.33633	-0.13429	C/C	3	4	9	5	6	5	9
22	13	m	1.06399	0.28222	0.22107	0.27571	0.36876	C/T	0	3	5	5	5	5	5
30	18	m	-0.09902	-0.75642	-0.79887	0.7137	0.16374	C/C	2	4	6	5	5	15	8
23	16	m	1.09822	2.3074	1.36931	1.82787	3.28031	C/T	16	4	11	7	5	11	8
25	12	m	1.50069	1.41986	0.48106	1.15364	0.79999	C/T	0	7	13	6	6	14	8
23	13	m	1.23684	1.72387	1.47531	1.24965	1.20382	C/T	8	6	13	9	5	17	10
20	12	m	-0.15716	0.63339	0.51625	0.42483	0.51561	C/C	4	5	7	8	5	12	8
19	13	m	1.23172	1.32366	1.40802	1.51517	0.76508	C/C	15	7	12	5	5	15	7
20	12	m	2.37783	1.36314	1.13234	1.87492	0.79128	C/C	12	5	10	9	6	11	10

23	15	m	0.36651	0.97385	0.76083	0.86647	0.98652	C/T	3	3	5	5	5	11	5
21	14	m	2.40143	2.71052	2.39762	3.4964	1.9725	C/T	13	9	5	6	5	7	5
27	16	m	1.49839	1.3663	1.30094	1.232584	0.48489	C/T	1	2	6	5	6	8	6
26	16	m	1.6316	1.39828	1.1753	1.63685	0.58122	C/C	5	4	8	5	5	12	7
28	16	m	-0.37715	0.18284	-0.26221	-0.11139	0.18417	C/C	7	3	6	7	5	14	6
20	14	m	0.5181	0.81138	0.48737	0.46449	-0.07693	C/C	6	8	8	7	5	16	6
25	16	m	1.27131	0.95544	1.03307	0.68475	0.38707	C/T	7	8	9	6	6	10	11
21	11	m	0.68626	1.15196	0.85637	0.98727	0.83999	C/C	2	7	13	5	5	8	5
26	16	m	0.34456	0.76847	0.74603	0.10337	0.87028	C/T	3	6	6	5	5	6	5
21	14	m	0.26661	0.73183	0.24132	0.07615	-0.03664	C/C	5	5	5	6	5	9	9
21	12	m	0.11661	-0.00005	-0.0947	0.27395	-0.14932	C/T	1	6	7	5	7	5	5
21	12	m	0.82546	0.70337	0.34062	0.68839	0.4616	C/C	2	3	7	6	5	13	6
23	13	m	0.65983	0.8906	0.18892	0.52096	0.30576	C/T	3	2	8	7	5	7	5
26	16	m	1.20861	1.48477	1.21389	1.47223	1.19808	C/C	4	4	5	5	5	6	5
20	12	m	-0.02876	0.3113	0.84068	0.34439	0.43575	C/C	15	5	16	5	6	10	10
20	12	m	-0.79335	1.20697	-0.10043	1.12232	0.70279	C/C	2	5	8	5	5	13	7
21	14	m	0.61298	1.57483	0.43298	0.67732	0.0672	C/T	6	6	8	6	5	9	7
25	14	m	1.17765	1.23298	0.98632	0.13077	0.87161	C/C	2	5	11	5	5	5	9
26	16	m	0.18823	0.42423	0.53974	0.81715	0.05402	C/T	4	4	12	5	5	6	10
24	14	m	0.48698	0.72574	0.48074	0.70529	0.52493	C/T	3	4	11	8	5	15	7
29	16	m	0.53594	0.75524	1.14352	0.40904	0.49253	C/C	6	8	13	7	9	12	6
20	13	m	1.59894	2.21287	1.55	1.85928	1.64788	C/C	12	7	11	11	11	16	6
22	14	m	1.45685	1.31307	0.93883	1.11266	0.12675	C/C	6	6	12	8	11	11	11
25	14	m	0.3051	0.39325	0.27323	0.31738	0.50072	C/T	4	5	19	11	10	17	12
28	16	m	0.7621	1.56351	1.13675	0.9519	1.20071	C/C	11	6	7	5	6	6	7
21	13	m	0.70406	0.685	0.78958	0.59402	0.34056	C/C	2	6	9	5	7	6	5
20	12	m	0.93994	1.50679	1.60481	1.38918	0.85496	C/T	2	3	10	5	10	6	10
24	15	f	1.85793	1.36094	1.12356	1.1658	0.43965	C/C	2	3	9	5	5	5	5
21	13	f	-0.05219	0.73357	0.61243	0.28522	0.97416	C/C	13	3	13	7	5	15	9
21	14	f	0.30077	1.54574	0.37671	0.00431	0.31724	T/T	9	3	8	14	7	14	12
21	14	f	-0.51321	-0.24499	-0.2229	-0.40144	-0.27768	C/T	6	7	12	8	6	13	6
21	14	f	0.98279	1.68304	1.00902	1.01545	1.0394	T/T	10	7	8	12	5	10	5
20	14	f	2.01361	2.48312	0.19437	1.25161	1.33114	C/C	5	6	8	10	5	14	5
20	12	f	-0.12618	0.69829	0.40281	0.38625	-0.31962	C/T	7	6	20	10	5	16	6
22	14	f	1.07412	1.41976	0.65376	1.18913	1.02387	C/C	4	6	6	5	5	6	5
25	16	f	1.66321	1.79533	2.6783	1.91094	2.09747	C/C	9	6	8	9	6	14	5
22	14	f	1.66378	1.79442	2.67887	1.9101	2.0972	C/C	10	8	5	15	11	13	5
24	15	f	1.00387	1.48322	0.85346	1.03359	1.7921	C/T	2	1	5	5	5	5	5
22	15	f	1.35804	2.09891	2.76167	2.14848	2.14719	C/C	8	4	7	5	5	10	5
22	14	f	-0.29864	-0.3522	-0.21661	-0.41024	0.39828	C/C	1	3	7	5	5	11	8
23	15	f	1.23473	1.45511	1.30133	0.91292	-0.02656	C/T	2	4	6	8	5	14	5
24	14	f	1.27413	1.50162	0.9628	0.86207	0.79259	T/T	6	7	6	6	5	#NULL!	9
20	12	f	0.67025	2.11991	1.94708	1.32172	1.48632	C/C	3	7	5	5	5	5	10
25	17	f	1.12792	1.85794	1.06542	1.61862	1.29839	C/C	4	4	5	5	5	5	5
25	18	f	-0.06854	0.29548	0.22273	0.21137	0.29069	C/C	3	3	5	5	5	5	6
20	12	f	0.73427	-0.75517	-0.46163	-0.51951	-1.1905	C/C	5	6	6	6	5	8	6
21	14	f	3.24436	3.15599	2.27847	2.46912	0.90696	C/C	10	3	20	22	8	24	11
22	12	f	0.48058	0.95874	0.04438	1.02372	0.41989	C/T	16	14	10	18	5	24	13
21	14	f	-0.02012	2.63286	2.71106	2.92517	2.20448	C/C	6	4	5	5	11	5	10
30	18	f	1.51245	1.33474	1.20123	1.31384	1.29759	C/C	11	10	5	5	5	10	5
30	18	f	1.88706	2.28657	1.94037	2.20607	1.9981	C/C	2	3	5	5	5	9	11
25	16	f	0.93048	0.71403	1.11493	0.59729	1.0011	C/C	3	3	7	5	5	6	9
29	17	f	0.54487	1.34206	0.8974	0.80651	-0.33137	C/T	3	8	6	5	5	15	9
28	16	f	0.03968	0.85355	0.55035	0.49589	0.85121	C/T	6	4	5	5	5	15	5
21	12	f	0.99417	1.15433	0.67829	1.13835	1.05807	C/C	7	5	8	8	7	9	8
28	15	f	-0.26344	2.19493	2.00111	1.41864	1.17277	C/T	15	12	5	5	5	15	11
21	15	f	1.37953	2.88778	2.2041	2.21301	0.50447	C/C	6	3	7	5	5	5	8
25	15	f	1.77155	2.25795	1.34473	1.71725	1.97399	C/C	3	4	7	5	5	12	5
22	15	f	-0.2791	-0.06503	-0.22379	-0.10033	-0.25002	C/C	2	7	5	5	5	5	9
22	15	f	0.42448	0.78523	1.38816	1.97322	0.73086	C/C	4	3	5	5	5	5	5
21	13	f	0.2818	1.05855	0.79812	0.78994	0.72926	C/C	3	8	5	6	10	8	6
27	18	f	1.24721	0.23464	0.40867	0.05078	-0.03397	C/T	2	4	6	5	5	5	6
22	15	f	-0.59257	-0.01213	0.12526	0.12776	0.22255	C/T	13	7	14	8	8	17	6
24	16	f	0.35417	0.95512	0.96778	0.73111	0.6	C/T	4	7	5	5	5	5	9
28	18	f	0.76105	1.45634	1.14005	1.20877	1.28622	C/T	6	7	7	5	6	5	5
25	16	f	0.30735	1.39276	0.66744	1.09436	0.54135	C/C	7	3	7	6	5	6	5
28	16	f	0.08963	0.06517	-0.06538	0.02323	0.26671	C/C	11	9	15	14	5	23	8
24	17	f	0.07103	-0.82576	0.13068	0.03208	0.16005	C/C	3	7	5	5	5	10	9
25	18	f	1.07293	0.05485	0.31945	-0.37072	0.13601	C/T	2	5	5	5	5	5	9
26	18	f	0.23708	-0.35039	0.09919	0.1177	-1.28418	C/C	5	9	8	7	5	9	7
25	12	f	1.14362	1.0856	0.30775	0.89515	-0.04402	C/C	7	8	10	14	5	17	14
25	16	f	1.06199	2.14261	1.70531	1.46041	1.84816	C/C	7	4	5	5	5	10	9
22	14	f	0.58376	0.90518	1.005656	0.8081	0.46456	C/T	10	6	10	9	5	11	5
20	12	f	0.60927	1.71501	1.08815	1.20646	0.47643	C/T	11	10	5	7	5	10	11
25	16	f	0.20354	2.87629	2.4139	2.3766	0.95645	C/T	5	2	6	5	5	9	5
20	12	f	1.1467	2.55033	2.05186	1.97387	1.9007	C/C	8	3	11	7	5	5	7
22	15	f	-0.03068	-1.22396	1.81679	1.67157	0.07922	C/C	4	2	5	5	5	10	5
21	12	f	1.64791	1.5853	1.72202	1.27987	1.22025	C/C	7	5	10	14	5	15	11
26	18	f	0.79842	1.01939	0.91461	0.80849	1.04995	C/C	1	3	5	5	5	17	5
23	16	f	-0.37121	-0.0783	-0.25192	0.07898	-0.25631	C/C	6	3	6	5	5	5	9
23	16	f	3.50329	2.8816	2.06174	1.95692	0.80492	C/C	4	6	5	5	7	8	8
23	16	f	0.29471	0.45599	0.55732	0.39358	0.20736	C/C	3	4	8	5	5	6	5
29	16	f	1.30352	1.83329	1.80068	1.28489	0.74346	C/C	10	5	5	5	5	13	6
29	18	f	0.06911	0.26285	0.22304	0.15632	0.51679	C/T	7	7	7	5	6	9	5
27	17	f	2.03657	1.95134	2.12543	1.50136	1.68732	C/C	7	4	5	5	5	11	6
26	16	f	2.03349	2.20398	2.63284	2.31023	2.22728	C/C	7	4	9	5	9	10	13

21	15	f	1.53503	1.59599	1.39694	0.68865	0.23681	C/T	0	6	6	5	5	8	5
29	15	f	0.95487	1.84384	1.66219	2.22347	1.53607	C/T	7	8	7	5	5	10	6
28	14	f	-1.13872	0.05385	0.10777	0.04561	-0.0806	C/C	11	7	7	6	5	8	5
25	18	f	0.11672	0.27316	0.88998	0.24336	0.14437	C/C	9	6	8	5	6	5	5
24	17	f	1.04604	1.17094	0.58161	0.74158	0.59876	C/C	6	6	5	5	5	11	9
24	18	f	1.10011	1.54131	1.36594	1.09655	0.7892	C/C	3	5	5	5	5	9	9
22	17	f	-0.65562	-0.05352	0.15947	-0.21151	0.25001	C/T	9	9	12	8	5	5	5
25	17	f	2.60586	4.00054	2.87974	3.00093	2.25239	C/T	3	4	6	5	5	16	7
22	16	f	2.21537	1.37428	1.32389	1.54586	1.07699	C/C	7	2	7	5	5	5	5
20	12	f	0.23604	0.9814	0.30597	1.42071	0.61941	C/C	8	5	5	6	5	7	7
20	12	f	0.38759	0.70342	0.48174	0.70168	0.23611	C/C	4	5	6	5	5	7	5
23	12	f	1.3311	1.20824	1.36785	1.0793	0.55121	C/T	7	3	5	5	5	8	5
23	12	f	0.89065	1.48002	1.14166	1.3009	1.03966	C/C	8	5	7	8	5	9	5
22	15	f	0.54373	0.92161	0.81648	0.61732	0.545	C/T	8	5	5	5	5	5	5
22	12	f	0.65692	0.57274	0.14038	0.40009	0.13692	C/C	7	6	8	6	5	12	5
28	18	f	1.14218	1.68002	1.2335	1.55725	0.90822	C/T	7	7	12	8	5	14	7
21	12	f	-0.01305	0.43131	0.53515	0.00911	0.26489	T/T	5	2	14	8	7	18	6
25	15	f	0.62024	0.85806	0.88838	0.72318	0.69969	C/C	5	5	8	5	5	7	6
22	15	f	0.02288	0.43515	0.4137	-0.09472	0.5475	C/T	1	4	9	13	6	8	6
28	16	f	1.08851	1.26779	1.07626	0.71213	0.51569	C/C	3	3	9	11	5	18	10
24	16	f	0.28748	0.91372	0.55192	0.63647	0.61933	C/C	6	5	5	5	7	7	6
25	16	f	1.13715	1.93158	1.39278	1.39916	1.72683	C/T	6	5	15	11	6	10	5
25	16	f	1.20748	1.3568	0.89667	1.16532	0.81085	C/T	2	8	6	5	5	8	5
27	16	f	0.72051	1.89037	1.66404	2.34323	2.23311	C/T	5	4	8	8	5	5	5
25	16	f	-0.05376	-0.18235	-0.26149	-0.65164	-0.20395	C/T	4	5	7	5	5	10	5
25	16	f	-0.07919	-0.01807	-0.20325	-0.2371	0.16029	C/C	6	7	11	9	5	12	6
24	15	f	0.91859	1.36515	1.19108	1.14651	0.74505	C/T	5	4	5	5	5	5	5
32	16	f	3.43858	4.16736	2.43073	2.98094	1.59504	C/C	5	5	10	7	6	9	5
32	16	f	-0.07236	0.48924	0.80539	0.53325	1.24534	C/T	3	5	10	5	9	5	5
26	16	f	0.40816	1.179	1.1106	0.82239	1.07264	T/T	6	5	17	14	16	8	11
21	15	f	0.49021	0.33417	0.37644	0.13002	0.13877	C/T	4	5	5	5	5	8	9
28	18	f	0.82508	0.739	0.81916	0.87931	0.71058	C/C	5	6	7	5	7	7	5
25	16	f	0.81869	0.85433	0.68354	0.59415	0.15739	C/T	9	9	7	8	7	8	5
24	15	f	-0.70656	1.9867	0.88617	-0.23596	1.01957	C/T	4	6	8	9	6	8	6
23	17	f	0.67208	0.46544	0.4265	0.28688	-0.58884	C/T	6	4	10	7	5	12	6
27	15	f	0.46565	1.31071	0.85952	1.06348	0.91937	C/T	3	7	5	6	6	7	5
25	18	f	0.90121	0.81804	0.80331	0.68738	0.42774	C/C	2	7	9	5	5	7	6
