# Supplementary material for: Influence of the 5-HT3A Receptor Gene Polymorphism and Childhood Sexual Trauma on Central Serotonin Activity
Source: PLoS One. 2015 Dec 23;10(12):e0145269. doi: 10.1371/journal.pone.0145269 (PMC4689356; doi:10.1371/journal.pone.0145269)
Supplement: S2 Table — (DOCX) [file pone.0145269.s002.docx]

| **S2 Table. The mean LDAEP magnitude in comparison with previous studies** | | | |
| --- | --- | --- | --- |
| **LDAEP study (n)** | **Healthy** | **Depression** | **Schizophrenia** |
| **Our study** | MEAN (μV/10 dB) | | |
| Whole (206) | 0.85 |  |  |
| CC (115) | 0.89 |  |  |
| T carriers (91) | 0.81 |  |  |
| CSA yes (61) | 0.90 |  |  |
| CSA no (145) | 0.84 |  |  |
| CC & CSA yes (36) | 1.07 |  |  |
| CC & CSA no (79) | 0.81 |  |  |
| T & CSA yes (25) | 0.67 |  |  |
| T & CSA no (66) | 0.87 |  |  |
| **Min et al.** |  |  |  |
| Whole (143) |  | 0.90 |  |
| Suicide attempter (11) |  | 1.01 |  |
| Non-suicide attempter (130) |  | 0.89 |  |
| **Wyss et al.** |  |  |  |
| Healthy control (13) | 0.98 |  |  |
| Schizophrenia (13) |  |  | 1.34 |
